# Everolimus Induced Acute Fibrinous and Organizing Pneumonia (AFOP): A Rare Case Report

**DOI:** 10.1155/crpu/8402905

**Published:** 2025-10-14

**Authors:** Theeb Osama Sulaiman, Abdullah Arshad, Fatima Al-Hattab, Mohammed Alkatib, Mona Al-Langawi, Mushtaq Ahmed

**Affiliations:** ^1^Pulmonology Department, Hamad General Hospital, Hamad Medical Corporation, Doha, Qatar; ^2^Medicine Department, Hamad General Hospital, Hamad Medical Corporation, Doha, Qatar

## Abstract

A variety of conditions have been associated with acute fibrinous and organizing pneumonia (AFOP), including autoimmune disorders, infectious agents, posttransplantation complications, and medications. Everolimus, an oral selective inhibitor of the mammalian target of rapamycin, is commonly used to manage metastatic malignancies such as breast cancer. Here, we describe a patient with a history of advanced breast cancer who developed AFOP secondary to everolimus, with the condition improving following discontinuation of the drug and initiation of steroid therapy.

## 1. Introduction

Acute fibrinous and organizing pneumonia (AFOP) was initially characterized by Beasley et al. in 2002 as a distinctive histopathological pattern marked by the presence of intra-alveolar fibrin balls and organizing pneumonia [1]. In 2013, the American Thoracic Society and the European Respiratory Society formally designated AFOP as a rare subtype of idiopathic interstitial pneumonia [2].

While the majority of AFOP cases are idiopathic, some are associated with autoimmune diseases, infections, posttransplantation complications, or medications. Several drugs have been documented to cause AFOP, including amiodarone, abacavir, bleomycin, sirolimus, and azacitidine [3]. We present a case of AFOP diagnosed by pulmonary histopathology in a patient with metastatic breast cancer who was receiving everolimus. Discontinuation of everolimus and initiation of corticosteroid therapy resulted in full clinical and radiographic resolution.

## 2. History of Presenting Illness

**Figure 1:**
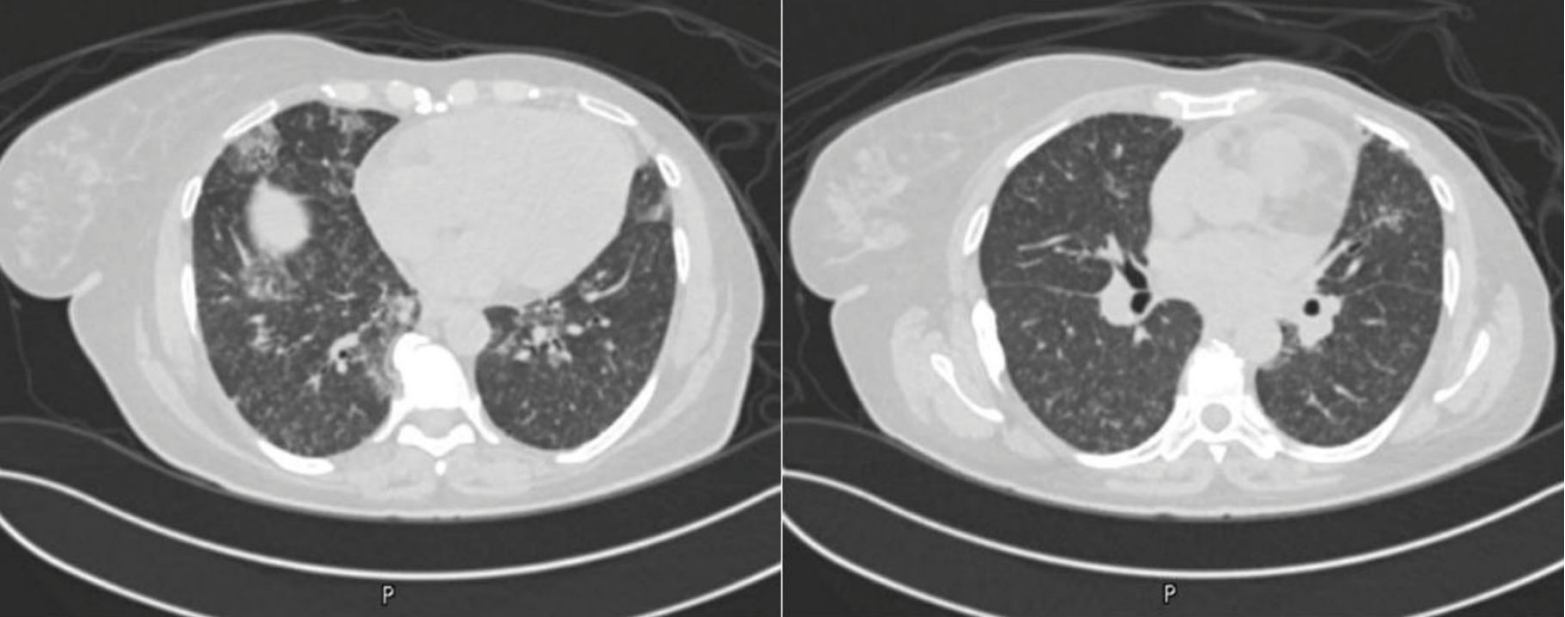
Chest CT scan showed diffuse numerous tiny nodules in both lung fields with areas of patchy GGOs and intralobular thickening more pronounced in both lower lobes.

A 54-year-old woman with a history of estrogen receptor (ER)- and progesterone receptor (PR)-positive, HER2-negative infiltrating ductal carcinoma of the left breast had undergone left mastectomy and adjuvant chemoradiotherapy. Several years later, she developed disease recurrence while on letrozole and palbociclib, prompting a regimen change to exemestane and everolimus in June 2021. During routine follow-up at the oncology clinic, a PET scan revealed numerous tiny random pulmonary nodules and areas of patchy septal and subpleural ground-glass opacities in both lower and middle lobes (Figure 1). The patient was referred to the pulmonary clinic for evaluation. She denied any respiratory symptoms, and there were no signs of connective tissue disease. She was a nonsmoker with no known exposure to toxins or pets.

**Figure 2:**
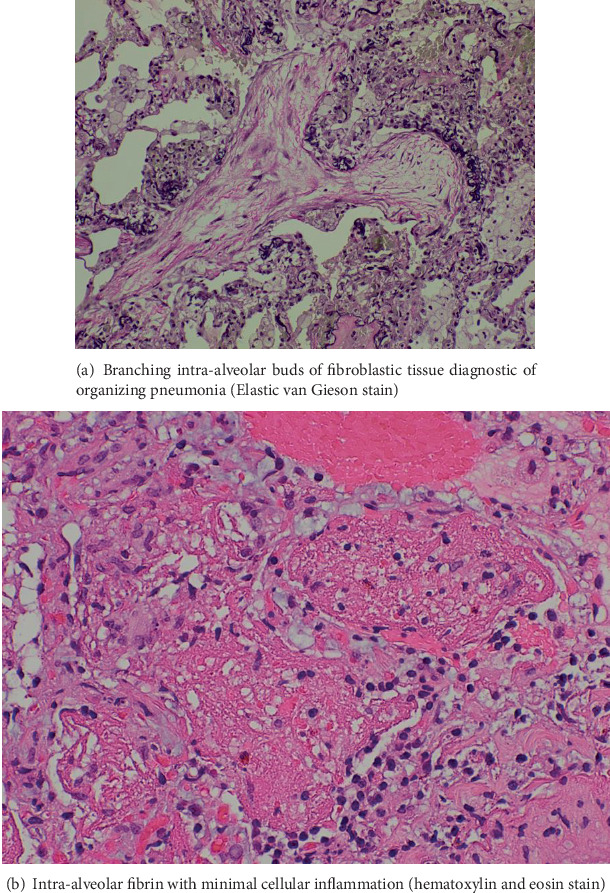
There is a patchy inflammatory process characterized by the presence of fibroblastic plugs filling the intra-alveolar spaces. Scattered fibrin balls are noted. Alveolar septae are remarkable for a few inflammatory cells, consisting mainly of lymphocytes and eosinophils. The appearances are those of an acute fibrinous and organizing pneumonia.

Bronchoscopy with transbronchial biopsy was performed. Bronchoalveolar lavage (BAL) was negative for infections, and differential cell count showed 13% lymphocytes. However, the biopsy did not yield a definitive diagnosis. A surgical lung biopsy via video-assisted thoracoscopic surgery (VATS) was subsequently performed, and histopathology revealed features consistent with AFOP (Figure 2). Before her scheduled pulmonary clinic follow-up, the patient presented to the emergency department with a short history of dyspnea, chest pain, and yellowish sputum production.

**Figure 3:**
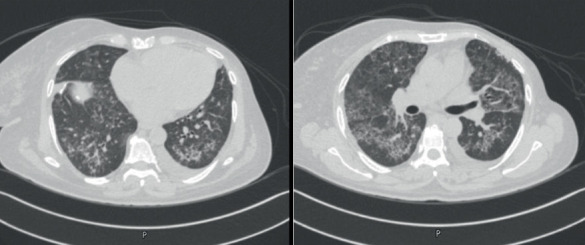
Chest CT scan demonstrated progression of GGOs compared to Figure 1.

**Figure 4:**
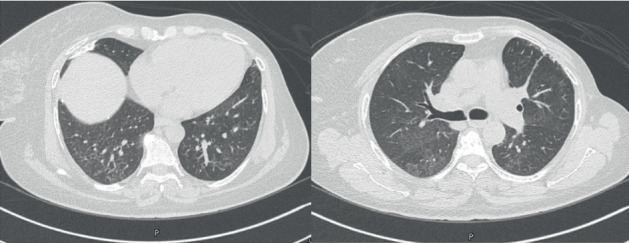
Chest CT scan showed appreciable regression of previously reported bilateral consolidation, ground-glass, and reticular pattern.

On presentation, she was afebrile and tachycardic with a heart rate of 127 beats/min, blood pressure of 129/83 mmHg, and oxygen saturation of 97% on 2 L/min nasal cannula. Pulmonary examination revealed decreased air entry at the lung bases and coarse crackles on the right. The remainder of the physical exam was unremarkable. Laboratory investigations showed the following: hemoglobin 9.1 g/dL, WBC count 13,200/*μ*L, platelets 543,000/*μ*L, serum creatinine 71 *μ*mol/L, urea 4.6 mmol/L, CRP 36 mg/L, and NT-proBNP 4929 pg/mL. Repeat chest imaging showed progression of pulmonary nodules and opacities (Figure 3). Echocardiography revealed a moderately reduced left ventricular ejection fraction of 37%. The patient was managed with antibiotics and furosemide, but symptoms persisted. Everolimus was discontinued by the oncology team, and the patient was started on prednisolone 30 mg daily for 4 weeks, followed by a taper of 5 mg per week. At the 2-month follow-up, she reported significant clinical improvement. A chest CT scan demonstrated regression of previously seen abnormalities (Figure 4). Steroids were discontinued after another 2 months, and she remained clinically stable with no recurrence at 14 months of follow-up.

## 3. Discussion

Everolimus is an oral selective mTOR inhibitor used in the treatment of various cancers, including breast and renal cell carcinoma. It is known to cause pulmonary toxicity such as noninfectious pneumonitis or interstitial lung disease (ILD) [4]. However, its association with AFOP is exceedingly rare, with only two documented cases in the literature [5, 6]. AFOP predominantly affects older adults (mean age approximately 66.4 years) and shows a male predominance [1, 7]. In one study involving 34 patients diagnosed with AFOP, 55.8% of cases were idiopathic, while 5.8% were attributed to drug-induced causes, including the use of afatinib and pembrolizumab [7]. Clinical presentation is nonspecific and may range from subacute symptoms to fulminant respiratory failure. In a study of everolimus-induced lung injury, the median onset of ILD was 57 days posttreatment initiation [8]. Due to nonspecific features, AFOP is often misdiagnosed [9].

Typical imaging findings include bilateral patchy consolidations (54%), ground-glass opacities (42%), and nodules (20%) [10]. Idiopathic AFOP more commonly presents with consolidation, while secondary AFOP (as in drug-induced cases) more frequently shows ground-glass changes. The gold standard for diagnosis is lung biopsy. In one series, surgical biopsy was required in most cases, as transbronchial biopsy had limited diagnostic yield due to patchy lesion distribution [11]. Histologically, AFOP is characterized by intra-alveolar fibrin balls without hyaline membranes or significant eosinophilic infiltration—distinguishing it from diffuse alveolar damage or cryptogenic organizing pneumonia [12]. BAL findings are usually non-diagnostic [10].

The cornerstone of management for drug-induced AFOP involves prompt withdrawal of the offending agent combined with systemic corticosteroid therapy. In one reported case of abacavir-induced AFOP, clinical improvement was achieved with drug discontinuation alone [13]. However, in another case of amiodarone-induced AFOP, resolution of symptoms required both cessation of the drug and initiation of methylprednisolone [14]. No standardized steroid regimen currently exists, but initial corticosteroid doses have ranged from 0.5 to 0.8 mg/kg/day, with an overall response rate of 94% [7]. Despite this, relapse occurred in 76% of cases within the cohort, with a median time to relapse of 163 days. Most relapses were successfully managed with steroid reinitiation, while only 9% of patients required additional immunosuppressive therapy. Other agents used successfully in AFOP include cyclophosphamide [15], mycophenolate mofetil [16], etanercept [17], indomethacin [18], intravenous immunoglobulin [19], infliximab, and tocilizumab [20]. Lung transplantation has been employed in cases progressing to irreversible respiratory failure [21]. Sirolimus has been reported to be safely administered in patients with everolimus-induced pneumonitis [22]. However, evidence regarding its safety and efficacy in cases of everolimus-induced AFOP remains lacking. The prognosis of AFOP ranges from complete recovery to death and depends on several factors, including the severity of lung injury, the nature of any underlying lung disease (such as ILD or chronic obstructive pulmonary disease), and the patient's overall health status. Reported mortality rates vary widely in the literature, from 6% to 53% likely due to AFOP's heterogeneous presentation [23, 24]. Further large-scale studies are needed to clarify its clinical course and outcomes, particularly in drug-induced cases.

## 4. Conclusion

The primary objective of this case report is to raise awareness of the exceedingly rare and not fully understood association between everolimus and AFOP.

Several therapeutic approaches are available for managing AFOP. In cases of drug-induced AFOP, the mainstay of treatment involves discontinuing the offending medication and initiating corticosteroid therapy.

## Data Availability

The datasets used and/or analyzed during the current study are available from the corresponding author upon reasonable request.

## References

[1] Beasley M. B., Franks T. J., Galvin J. R., Gochuico B., Travis W. D. (2002). Acute Fibrinous and Organizing Pneumonia. *Archives of Pathology & Laboratory Medicine*.

[2] Travis W. D., Costabel U., Hansell D. M. (2013). An Official American Thoracic Society/European Respiratory Society Statement: Update of the International Multidisciplinary Classification of the Idiopathic Interstitial Pneumonias. *American Journal of Respiratory and Critical Care Medicine*.

[3] Arnaud D., Surani Z., Vakil A., Varon J., Surani S. (2017). Acute Fibrinous and Organizing Pneumonia: A Case Report and Review of the Literature. *American Journal of Case Reports*.

[4] Mizuno R., Asano K., Mikami S., Nagata H., Kaneko G., Oya M. (2012). Patterns of Interstitial Lung Disease During Everolimus Treatment in Patients With Metastatic Renal Cell Carcinoma. *Japanese Journal of Clinical Oncology*.

[5] Gomes R., Padrão E., Dabó H. (2016). Acute Fibrinous and Organizing Pneumonia. *Medicine (Baltimore)*.

[6] White D. A., Camus P., Endo M. (2010). Noninfectious Pneumonitis After Everolimus Therapy for Advanced Renal Cell Carcinoma. *American Journal of Respiratory and Critical Care Medicine*.

[7] Onishi Y., Kawamura T., Higashino T., Mimura R., Tsukamoto H., Sasaki S. (2021). Clinical Features of Acute Fibrinous and Organizing Pneumonia: An Early Histologic Pattern of Various Acute Inflammatory Lung Diseases. *PLoS One*.

[8] Saito Y., Kunugi S., Suzuki Y. (2013). Granuloma-Forming Interstitial Pneumonia Occurring One Year After the Start of Everolimus Therapy. *Internal Medicine*.

[9] Santos C., Oliveira R. C., Serra P. (2019). Pathophysiology of Acute Fibrinous and Organizing Pneumonia - Clinical and Morphological Spectra. *Pathophysiology*.

[10] Chen H., Kuang Y., Huang X. (2021). Acute Fibrinous and Organizing Pneumonia: Two Case Reports and Literature Review. *Diagnostic Pathology*.

[11] Kuza C., Matheos T., Kathman D., Heard S. O. (2016). Life After Acute Fibrinous and Organizing Pneumonia: A Case Report of a Patient 30 Months After Diagnosis and Review of the Literature. *Journal of Critical Care*.

[12] Chaudhary N. M., Crowell W., Katzman J. H., Klinkova O., Greene J. (2021). Acute Fibrinous and Organizing Pneumonia Case Report and Review: Distinguishing AFOP From Similar Organizing Pneumonias. *Journal of Family Medicine*.

[13] Yokogawa N., Alcid D. V. (2007). Acute Fibrinous and Organizing Pneumonia as a Rare Presentation of Abacavir Hypersensitivity Reaction. *Aids*.

[14] Piciucchi S., Dubini A., Tomassetti S., Casoni G., Ravaglia C., Poletti V. (2015). A Case of Amiodarone-Induced Acute Fibrinous and Organizing Pneumonia Mimicking Mesothelioma. *American Journal of Respiratory and Critical Care Medicine*.

[15] Damas C., Morais A., Moura C. S., Marques A. (2006). Acute Fibrinous and Organizing Pneumonia Pneumonia Aguda Fibrinosa e Organizante. *Revista Portuguesa de Pneumologia*.

[16] Bhatti S., Hakeem A., Torrealba J., McMahon J. P., Meyer K. C. (2009). Severe Acute Fibrinous and Organizing Pneumonia (AFOP) Causing Ventilatory Failure: Successful Treatment With Mycophenolate Mofetil and Corticosteroids. *Respiratory Medicine*.

[17] Simmons G. L., Chung H. M., McCarty J. M. (2017). Treatment of Acute Fibrinous Organizing Pneumonia Following Hematopoietic Cell Transplantation With Etanercept. *Bone Marrow Transplantation*.

[18] Zhou C. X., Tang T. T., Huang L. J. (2016). Methylprednisolone Combined With Low-Dose Indomethacin Treating Acute Fibrinous and Organizing Pneumonia After a Surgical Resection of Rectal Adenocarcinoma: A Case Report and Literature Review. *European Review for Medical and Pharmacological Sciences*.

[19] Kashif M., Arya D., Niazi M., Khaja M. (2017). A Rare Case of Necrotizing Myopathy and Fibrinous and Organizing Pneumonia With Anti-EJ Antisynthetase Syndrome and SSA Antibodies. *American Journal of Case Reports*.

[20] Acute Fibrinous and Organizing Pneumonia (AFOP) in Lung Transplant Recipient: A Case Report of Successful Treatment With Infliximab and Tocilizumab.

[21] Campisi A., Dell’Amore A., Bertolaccini L. (2020). Urgent Lung Transplantation in Acute Fibrinous and Organizing Pneumonia: A Sliding Door or a New Perspective?. *General Thoracic and Cardiovascular Surgery*.

[22] Wang H., Chen C., Hu D., Tian X., Zhang H., Xu K.-F. (2024). Sirolimus Safely Used in a Patient After Everolimus-Induced Pneumonitis: A Case Report [Abstract]. *American Journal of Respiratory and Critical Care Medicine*.

[23] Kim M. C., Kim Y. W., Kwon B. S. (2022). Clinical Features and Long-Term Prognosis of Acute Fibrinous and Organizing Pneumonia Histologically Confirmed by Surgical Lung Biopsy. *BMC Pulmonary Medicine*.

[24] Lu Y., Zheng W., Cao W., Yang X., Zhao L., Chen Y. (2022). Acute Fibrinous and Organizing Pneumonia in a Patient With Sjögren’s Syndrome and Legionella Pneumonia: A Case Report and Literature Review. *BMC Pulmonary Medicine*.

